# Laparoscopic treatment of abdominal unicentric castleman’s disease: a case report and literature review

**DOI:** 10.1186/s12893-017-0238-6

**Published:** 2017-04-12

**Authors:** Umberto Bracale, Francesco Pacelli, Marco Milone, Umberto Marcello Bracale, Maurizio Sodo, Giovanni Merola, Teresa Troiani, Enrico Di Salvo

**Affiliations:** 1 Department of Surgical Specialities and Nefrology, University “Federico II” of Naples, Via Pansini, 5, Naples, 80100 Italy; 2 Department of Clinical and Experimental Medicine ‘F. Magrassi’, Second University of Naples, Naples, Italy

**Keywords:** Castleman’s disease, Case report, Mesenteric tumor, Laparoscopy

## Abstract

**Background:**

Castleman’s disease is a rare lymphoproliferative disorder of unknown etiology that most commonly presents as a mediastinal nodal mass. It is exceptionally uncommon for Castleman’s disease to present in the mesentery and, only 53 cases have ever been described in the literature. Standard treatment for this lymphoproliferative disorder involving a single node is a complete “en bloc” surgical resection which has proven to be a curative approach in almost all cases without recurrence after 20 years of follow up. All 53 reported cases of mesenteric Castleman’s disease, except one, were treated with laparotomy.

**Case presentation:**

We report on a case of mesenteric Castleman’s disease localized in the mesentery which is the second reported case if its kind and was treated by a laparoscopic-assisted procedure. Our female patient had an uneventful postoperative course and was discharged in the 5^th^ post-operative day. No signs of recurrence were present as evidenced by physical examination and total body CT scan 24 months after the operation. We compare our case with the other reported cases in which Castleman’s disease presented as an isolated mass in the abdomen.

**Conclusion:**

Although a rare disease, Unicentric Castleman’s disease should always be considered when a solid asymptomatic abdominal mass is occasionally presented. The laparoscopic approach (LA) allows for the achievement of better results than open surgery, including a reduction in postoperative pain and length of hospital stay. In cases of masses of an uncertain nature, LA must be considered the last diagnostic tool and the first treatment one.

## Background

Castleman’s disease (CD) is a rare and benign lymphoproliferative disorder that can involve single (unicentric) or multiple lymph nodes (multicentric). It can be classified into three histopathological patterns: hyaline-vascular (HV) type, plasma cell (PC) type and mixed variant [1, 2]. Usually the HV type appears more frequently as a unicentric localization whereas the PC type and mixed variant are mostly multicentric [3, 4]. Although Unicentric Castelman’s Disease (UCD) can affect any nodal station, a typical localization of the disease is in the mediastinum (70% of cases). Mesenteric localization of UCD is very rare and a differential diagnosis between UCD and other disorders is very difficult to achieve. [5]. The Laparoscopic approach (LA) represents the gold standard treatment in many abdominal diseases [6]. It provides an alternative to an open approach that may reduce postoperative pain, postoperative complications and result in a shorter hospitality stay.

The aim of this report is to describe a case of UCD localized in the transversal mesocolon treated by LA at our center. We also carried out a Literature Review about Laparoscopic treatment of Abdominal UCD which is reported herein.

## Case presentation

**Fig. 1:**
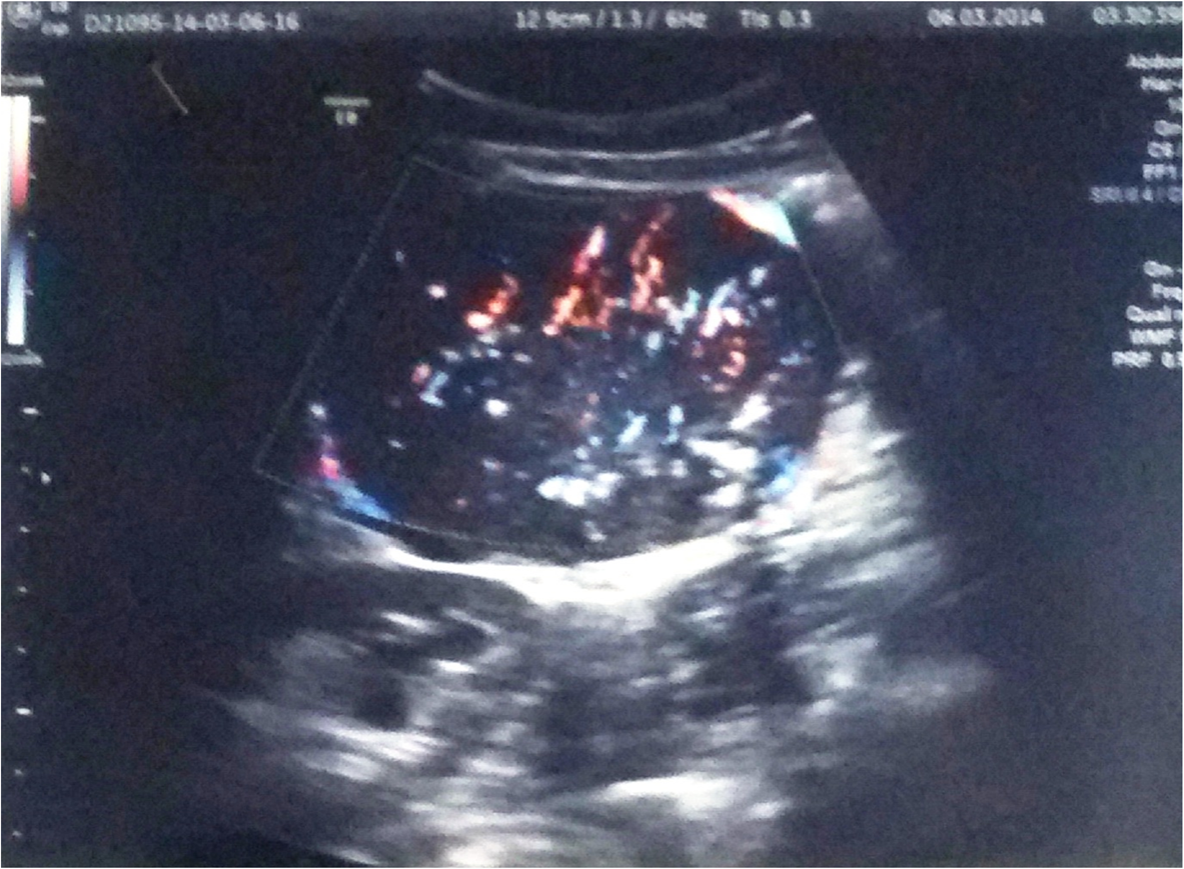
US scan of Lesion

**Fig. 2:**
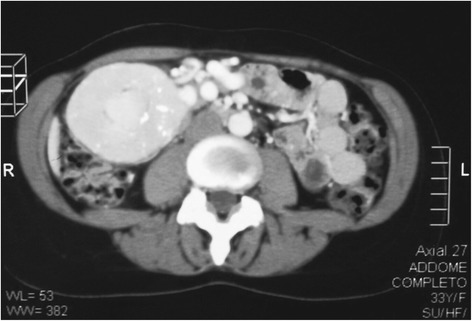
CT scan showing a solid inhomogeneous mass, with inner calcifications

**Fig. 3:**
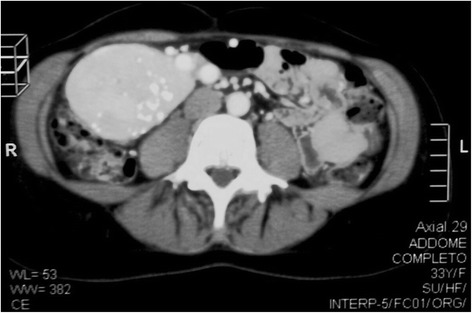
CT scan showing a solid inhomogeneous mass, with inner calcifications

A 33 year-old female patient was admitted to our General Surgery Department in March of 2014 due to the presence of a palpable mass in her right abdominal flank and dyspeptic symptoms. She had been a smoker for about 15 years and was in good general status with a Body Mass Index of 20.5. She reported the 4 year presence of a painless mass which had been revealed upon a first abdominal wall ultrasonography (US) that showed in the right para-umbilical region a solid slightly hyperechoic mass of 1. 5 cm diameter, reported as consistent with a lipoma. She had an operative history of umbilical hernia repair without mesh. After 4 years of wellness, she repeated an abdominal US which revealed a defined solid lesion measuring 8.2 × 6.1 × 6.8 cm under the inferior hepatic edge and close to the inferior cava vein and the inferior pole of the right kidney (Fig. 1). The subsequent abdominal computed tomography (CT) showed a solid heterogeneous mass with inner calcifications measuring 9 × 7 x 6 cm, hypervascular and well circumscribed from the pancreatic head, liver and inferior pole of the kidney (Figs. 2 and 3). No bowel obstruction and no other masses or lymphadenopathy were observed. A physical examination revealed the presence of a palpable, mobile mass in the right abdominal quadrant without tenderness. No other lymph node enlargements were found. Pre-operative blood tests only showed elevated the CA 125 marker (81,4 U/ml, with normal value < 35).

The patient underwent a laparoscopic procedure with three 11-mm ports in standard position for a right colectomy. Laparoscopic exploration showed a mass in the context of the transversal mesocolon, connected to the middle colonic vessels and ahead the duodenum. The procedure started with the opening of the gastro-colic ligament using Ultracision® (Ethicon Endo-Surgery Inc, Cincinnati, OH, USA). The hepatic flexure was mobilized respecting Gerota’s and Toldt’s Fascias. The entire mass was well isolated laparoscopically and completely removed through a xifo-umbilical incision. The procedure was conducted in 120 min with no intraoperative complications.

Macroscopically the mass measured 9 × 8 × 4 cm in size, surrounded by a thick whitish fibrous capsule. The histopathological report referred an enlarged lymph node with multiple lymphoid follicular, fibroblastic proliferation, multiple fibrotic septa and hyalinised vessels. An Immunochemistry study showed dendritic cells (CD 21+, CD 23+) and small mantle-zone lymphoid cells (CD20^+^, bcl-2^+^). The final diagnosis was of UCD, hyaline-vascular subtype (UCD-HV). The patient had an uneventful postoperative course and was discharged on the 5^th^ post-operative day. At this time no signs of recurrence are present by physical examination nor by total body CT scan 24 months after the operation.

## Discussion and conclusions

Castleman’s disease was described for the first time in 1954 by Benjamin Castleman, a pathologist at Massachusetts General Hospital, as an uncommon lymphoproliferative disorder and subsequently in 1956 as a benign, localized thymoma-like enlargement involving hyperplastic lymph nodes in the anterior mediastinum [7, 8]. Earlier synonyms of CD included “angiofollicular lymph node hyperplasia”, “giant cell lymph node hyperplasia”, “follicular lymphoreticuloma”, “lymphoid hamartoma” and “benign lymphoma”.

### Incidence and classification

The prevalence of CD has not been estimated, but it has been calculated that the number of cases in the United States ranges from 30.000 to 100.000 [9]. Its incidence rate has not been reported in literature, although CD appears to be more common in the Asian population [10].

A commonly used system to classify the heterogeneity of CD was proposed by McCarty et al. in 1955 [11]. Based on clinical and radiological characteristics, CD can be classified as unicentric (unifocal) or multicentric (MCD) form, depending on the number of lymph nodes involved.

UCD represents the most common form (>90%) of CD and is asymptomatic in over half of cases. Sometimes, when the lesion is large enough, compressive or constitutional symptoms may be present. It tends to occur in the third and fourth decade of life with a slight female predominance with a median age of 35 years [12, 13]. The age of the patient reported in this case is in line with the average age of all other patients with UCD reported in the literature.

### Pathologic mechanism

The pathophysiological basis of Castleman’s disease is still unclear. However, chronic low-grade inflammation, immunodeficiency status and dysregulation autoimmunity have been proposed as likely pathogenic mechanisms. The critical role of inflammatory mediators such as interleukin 6 (IL-6) or interleukin 10 (IL-10) and human herpes virus 8 (only in Multicentric variant) has been well demonstrated in preclinical animal models [14]. Dysregulation and overexpression of IL-6 stimulate hepatocytes to produce acute phase proteins which increase the levels of the hepcidin hormone, which correlates with anemia. IL-6 also stimulates B-cells and blood vessel proliferation promoting the overexpression of the vascular endothelial growth factor and the neoangiogenesis. Interestingly, a recent study has demonstrated that hyaline-vascular Castleman’s disease is often a monoclonal proliferation, consisting most likely of lymph node stromal cells [15].

### Histological features

CD can be classified into three histopathological patterns: a hyaline-vascular (HV) type, a plasma cell (PC) type and a mixed variant. Usually it is the HV type that represents 80–90% of cases and appears more frequently as unicentric localization (UCD) whereas the PC type is mostly multicentric (MCD) and accounts for only 10–20% of cases.

In the HV variant, lymph nodes involved in the disease, show increased numbers of lymphoid follicles that exhibit features of “regression”: a term referring to a predominance of dendritic cells relative to lymphocytes within germinal centers and consequent rearrangement of mantle zones, known as an “onion ring pattern”. Also, an increased number of small hyalinized vessels between and within follicles, named “lollipop follicles”, results in obliteration of medullary sinuses. In the unicentric localization the average size of lymph nodes is very wide, ranging from 1 to 12 cm. The lesion size reported in this case is consistent with those reported in literature.

### Location

UCD most frequently affects lymphoid tissues of the thorax (70%) neck (15%), abdomen-pelvis (12%) and axilla (3%). The location of the disease in mesentery is rare and usually associated with multicentric form. In a recent case report and literature review [16], only 53 cases of mesenteric UCD were reported worldwide. All these cases except one were treated with a laparotomy. To the best of our knowledge our case is the second reported of case of UCD located in the mesentery and treated by a laparoscopic-assisted procedure.

### Clinical manifestations

UCD usually is identified without symptoms at diagnosis and can be discovered incidentally in chest or abdominal-pelvic imaging performed for other reasons. The patients may present symptoms related to the compression of adjacent organs. Dyspnea, cough, hemoptysis, and chest pain can be present in thoracic disease whereas vomiting, postprandial discomfort and abdominal or lumbar pain in abdominal-retroperitoneal disease [17–19]. Therefore, because there are no specific symptoms and clinical presentation can vary greatly, a diagnosis of UCD based only upon clinical features is difficult.

In our case the patient only complained about abdominal discomfort. Laboratory studies show normal levels of cytokines (C-reactive protein, IL-6) in the absence of anemia or thrombocytopenia with a normal T and B cell count.

### Diagnosis

UCD diagnosis is based on clinical evaluation, which includes patient history, laboratory and radiological findings, but a final diagnosis can be achieved only by careful histological and immunohistochemical examination. Therefore, preoperative diagnosis is often not achievable. The laboratory evaluation of patients with UCD includes a complete cell blood count and metabolic panel, inflammatory markers, albumin and Human Immunodeficiency Virus (HIV) and Human Herpes Virus (HHV)- 8 tests. Plasmatic elevated levels of cytokines such as IL-6 and IL −10, can lead to its diagnosis but are not routinely recommended in clinical practice. As our patient had no abnormal laboratory tests, it was difficult to make the diagnosis of UCD on the basis of these analyses alone. Moreover, a circulating HHV-8 search resulted negative in our patient.

The UCD diagnosis of certainty is usually obtained by performing an excisional biopsy of the pathological lymph nodes. In the case of a less accessible disease core needle biopsy (FNAB) is preferred to fine needle aspiration (FNAC), which is commonly not diagnostic. In fact, a preoperative FNAB or FNAC is not recommended because of the difficulty of achieving an adequate amount of tissue, the possibility of spreading tumor cells and the risk of severe bleeding in hypervascular mass. In all cases reviewed in the literature, all authors performed a preoperative Computed Tomography (CT) scan, often proceeded by an Ultrasonography (US) and followed by Magnetic Resonance Imaging (MRI), Positron Emission Tomography (PET) or Fine Needle Aspiration Biopsy (FNAB). A preoperative diagnosis of CD was not suspected in any of the cases [20, 21]. This is consistent with our clinical case in which a definitive preoperative diagnosis was not obtained. In particular, although endoscopic or ultrasound-TC guided fine needle biopsy is recommended by many authors, severe bleeding risk in hypervascular mass should be taken into account. Based on all these considerations, we do not perform a preoperative cytological diagnosis due to the risk of bleeding.

Although UCD is a not a malignant condition, different malignancies and other diseases have been associated with it.[22]. Non-Hodgkin lymphoma and amyloidosis have been reported in approximately 18% of patients with MCD, as well as in patients with UCD [23]. Paraneoplastic pemphigus is also associated with UCD in about 20% of cases and characterized by an increased risk of lymphoma [24]. Lymphoma, lymph node metastasis, paraganglioma, gastrointestinal stromal tumor (GIST), ectopic pheochromocytoma, leiomyoma and leiomyosarcoma, liposarcoma, fibrosarcoma can be included in differential diagnosis with mesenteric UCD, especially for female patients [25]. Moreover, the differential diagnosis must be performed with other causes of lymphadenopathy such as tuberculosis, luetic lymphadenitis, abscess, sarcoidosis, HIV and toxoplasmosis. Because the radiological findings for UCD are unspecific, the preoperative radiologic differential diagnosis of mesenteric disease most commonly includes hypervascular mesenchymal tumors such as GIST, neurogenic tumors such as ectopic pheochromocytoma, carcinoids or pancreatic cancer. Due to the face that many patients show very similar radiologic features, a differential diagnosis is very difficult to arrive at.

Unfortunately, the characteristics detectable for diagnostic tools (US: CT scan, MRI or PET) are not conclusive for CD even if Malara et al. described in detail the US and CT features of mesenteric UCD [26]. Most cases of abdominal UCD cannot be visible on radiographs unless they are massive or have calcifications. Abdominal UCD usually presents as a homogeneus and hypoechoic solitary mass by US. In contrast, abdominal US of our patient showed heterogeneity of the mass, perhaps due to its large size. Homogeneity with intense contrast enhancement reflecting hypervascularity of the lesion is a characteristic finding at CT of abdominal UCD. Mesenteric UCD commonly appears at CT as a well-defined single mass of soft tissue without satellite nodules or surrounded by normal lymphadenopathy [27, 28]. UCD usually results positive on fluorodeoxyglucose PET [29].

### Therapy

The standard treatment for UCD regardless of histological type (whether HV or PC), is a complete “en bloc” surgical resection, which is a curative approach in almost all cases without recurrence after 20 years of follow up [30]. A subtotal resection presents a low recurrence rate and can be cured by re-excision. In Table 1 we report on all cases of abdominal UCD treated laparoscopically which have been published in the literature [31–42]. In five cases the disease was localized in extralymphatic tissues such as pancreas, liver, spleen and the adrenal gland. In these cases, pancreatic cancer, splenic abscess, an accessory spleen, hepatocellular carcinoma and pheochromocytoma were suspected preoperatively. In contrast, lymphatic tissue localizations were defined preoperatively as adnexal mass, lymphoma or metastatic disease. Our preoperative diagnosis was consistent with that reported by Ohta et al. who performed a laparoscopic ileal resection suspecting a GIST localization [35]. Our case was resolved without bowel resection because of the presence of an adequate dissection plane.

**Table 1:** Studies about laparoscopic treatment of abdominal castelman’s disease

	Author	Localization	Sex	Symptoms and/or Signs	Preoperative Study	Suspected Diagnosis	Positive Markers	Surgical Technique	Istology
1.	Lee J. [9]	Pelvic	F	None	CT, TVUS	Adenexal Mass	None	Single-Port Laparoscopic Mass Excision	7-cm HV Type
2.	Miyoshi H. [10]	Liver VI Segment	F	Epigastric Pain	US, CT, MRI, PET, EGDS, Colonoscpy	HCC	None	Laparoscopic Assisted Right Lobectomy	2-cm HV Type
3.	Jang S.Y. [11]	Hepatoduodenal Ligament	F	Right Quadrant Pain	CT, MRI, SA	Exophytic HCC	None	Totally Laparoscopic Resection	3-cm HV Type
4.	Bauters A. [12]	Omentum	F	None	CT, MRI		Fibrinogen	Totally Laparoscopic Resection	3-cm PC and HV Type
5.	Ohta M. [13]	Jejunal Mesentery	F	None	US, CT, MRI	Duodenal Gist	None	Laparoscopic Assisted Resection	7-cm HV Type
6.	Lee H.J. [14]	Spleen	M	Abdominal Pain, Fever, Diarrhea	CT	Lymphoma, Splenic Hamartoma or Abscess	CRP, ESR	Laparoscopic Splenectomy	7-cm HV Type
7.	Cecka F. [15]	Pancreas	F	Epigastric pain	CT, EUS, FNAB	Gastric GIST, Pancreatic Tumour	None	Laparoscopic Pancreatic Resection	4-cm HV Type
8.	Martin A.K. [16]	Right Retroperitoneal Mass	M	Nausea and Vomiting	EUS biopsy; CT-Pet	Lymphoma, Metastatic Disease, Extra-Adrenal Pheocromocytoma, Testicular Cancer.	None	Totally Laparoscopic Resection	5,5 cm HV Type
9.	Brusciano L. [17]	Posterior Surface of Abdominal Wall	M	Palpable Mass	CT		None	Totally Laparoscopic Resection	5 cm PC Type
10.	Corcione F. [18]	Lower Splenic Pole	M	Recurrent Palpitation and Vague Abdominal Pain	US, CT	Accessory spleen	None	Totally Laparoscopic Resection	5-cm HV Type
11.	Otto M. [19]	Right Adrenal Gland	M	None	US, CT	Adrenal Gland, Pheocromocytoma	None	Laparoscopic Adrenalectomy	4,5 cm HV Type
12.	Rosado R. [20]	InterAorto-Caval Mass	F	Anemia	CT, FNAB			Converted Laparoscopy	6.7-cm PC and HV Type

*F* Famale, *M* Male; *CT* Computed Tomography, *TVUS* TransVaginal UltraSound, *HV* Hyaline-Vascular Type, *HCC* HepatoCellular Carcinoma, *MRI* Magnetic Resonance Imaging, *PET* Positron Emission Tomography, *EGDS* Esophago-Gastro-Duodenoscopy, *SA* Selective Angiography, *PC* Plasmacell Type, *CRP* C-Reaction Protein, *ESR* Erythrocyte Sedimentation Rate, *EUS* Endoscopic UltraSound, *FNAB* Fine Needle AgoBiopsy, *US* UltraSound

As shown in Table 1, all the cases of abdominal UCD treated with LA were completed laparoscopically, with the exception of one [42]^.^ In this case, the mass was adherent to the cava vein and so the authors converted the procedure to obtain safer vascular control. In the other cases, surgeons performed a mass removal laparoscopically or an "en bloc" resection of the organ in which was it contained. All procedures were bloodless. No other intraoperative or postoperative complications occurred and patients were discharged earlier (range 1–5 days). Based upon this positive experience all the Authors concluded that laparoscopy could be a safe and effective procedure for the treatment of UCD.

We opted for a LA to ensure the patient the typical benefits of the technique. Usually, we remove the specimen through a Pfannenstiel incision. In this case, both for the size of the lesion and for the presence of a previous umbilical incision, we opted for a xipho-umbilical incision as reported in the literature for more complex Gastric procedures.

The literature review suggests that radiotherapy can be a more favorable treatment to UCD than invasive surgical resection with a minimal complication rate and good prognosis [43, 44]. Complete clinical and radiologic resolution of UCD is consistently documented in other articles. Intensity-modulated radiation therapy is better than three-dimensional conformal therapy due to its reduction of the dose gradient and toxicity to the surrounding normal tissue [45]. De Vries et al. demonstrated that neoadiuvant radiotherapy used to downsize advanced unresectable UCD in order to achieve a radical excision could be a possible strategy of treatment [26].

When surgical resection and radiotherapy are impossible, partial resection followed by clinical observation alone may be useful and can result in a lengthy remission.

In conclusion, although a rare disease, UCD should always be considered when a solid asymptomatic abdominal mass is incidentally found. The pelvis and retroperitoneum are USDs most frequent sites, and a correct pre-operative study and surgical timing can lead the patient to a full recovery. Moreover, based upon our experience we retain that a laparoscopic approach leads to better results than open surgery as it reduces postoperative pain and limits the length of hospital stay. In cases of an uncertain nature mass, LA must be considered as the last diagnostic tool and the first treatment one.

## Abbreviations

CT: Computed tomography
FNAB: Fine needle AgoBiopsy
FNAC: Fine needle aspiration
GIST: gastrointestinal stromal tumor
HHV: Human herpes virus
HIV: Human immunodeficiency virus
HV: Hyaline-vascular type
LA: Laparoscopic approach
MCD: Multicentric castelman’s disease
MRI: Magnetic resonance imaging
PC: Plasma cell type
PET: Positron emission tomography
UCD: Unicentric castelman’s disease
US: UltraSound scan
